# A generic nomogram for multinomial prediction models: theory and guidance for construction

**DOI:** 10.1186/s41512-017-0010-5

**Published:** 2017-04-10

**Authors:** Maarten van Smeden, Joris AH de Groot, Stavros Nikolakopoulos, Loes CM Bertens, Karel GM Moons, Johannes B. Reitsma

**Affiliations:** 1 Julius Center for Health Sciences and Primary Care, University Medical Center Utrecht, Heidelberglaan, Utrecht, 100 Netherlands; 2 Department of Obstetrics and Gynaecology, Erasmus MC, Rotterdam, Netherlands

**Keywords:** Prediction, Nomogram, Graphical presentation, Multinomial outcomes, Logistic model, Scoring chart

## Abstract

**Background:**

The use of multinomial logistic regression models is advocated for modeling the associations of covariates with three or more mutually exclusive outcome categories. As compared to a binary logistic regression analysis, the simultaneous modeling of multiple outcome categories using a multinomial model often better resembles the clinical setting, where a physician typically must distinguish between more than two possible diagnoses or outcome events for an individual patient (e.g., the differential diagnosis). A disadvantage of the multinomial logistic model is that the interpretation of its results is often complex. In particular, the calculation of predicted probabilities for the various outcomes requires a series of careful calculations. Nomograms are widely used in studies reporting binary logistic regression models to facilitate the interpretation of the results and allow the calculation of the predicted probability for individuals.

**Methods and results:**

In this paper we outline an approach for deriving a generic nomogram for multinomial logistic regression models and an accompanying scoring chart that can further simplify the calculation of predicted multinomial probabilities. We illustrate the use of the nomogram and scoring chart and their interpretation using a clinical example.

**Conclusions:**

The generic multinomial nomogram and scoring chart can be used irrespective of the number of outcome categories that are present.

## Background

The use of multinomial logistic regression modeling has been encouraged to study multiple unordered outcome categories (and possibly their combination) simultaneously [1, 2]. The multinomial logistic model can be considered to be an extension of the popular binary logistic regression model, which is often used in the presence of two mutually exclusive outcome categories. More specifically, multinomial logistic regression analysis can be viewed as a series of binary logistic regression analyses where one of the outcome categories is the reference category in each binary sub-model.

Researchers often collapse multiple outcome categories into a single binary (composite) outcome and use binary logistic regression models to analyze their data. Consequently, potentially important information about the different outcome categories is lost. It can also be argued that a binary outcome often does not accurately reflect clinical practice, where physicians commonly have to make decisions while considering more than two relevant choices. For instance, physicians often consider the presence of differential diagnoses (and prognoses) for an individual patient simultaneously [1].

One of the key reasons why researchers might refrain from multinomial logistic regression analysis is that the results from these models are more complex to interpret and more elaborate than results from a binary logistic regression analysis. In the multinomial context, regression coefficients are estimated for each binary sub-model reflecting the relation of covariates to one outcome category relative to the reference category. The number of estimated parameters quickly increases with additional outcome categories considered. The large amount of information from multinomial models can easily overwhelm researchers and clinicians. In addition, when using the multinomial logistic model for estimating probabilities for individual patients, the computation involved for the various outcomes requires a series of careful calculations.

Nomograms found many applications in the reporting of binary logistic regression models (for recent examples, see [3, 4]). A nomogram can not only improve insights of clinicians into the results of a logistic model, it can also be used to arrive at a predicted probability of outcome(s) of interest that is (are) tailored to the profile of an individual patient in a graphical manner. Nomograms can thus facilitate clinical decision making during clinical encounters. So far, nomograms have been used primarily for improving the reporting of models with only two outcome categories. We are aware of one recent paper that reported on the construction and one on the application of a nomogram for multinomial models. However, the focus of these papers was limited to constructing a nomogram for a limited number of outcome categories [5] and the reporting of results of one specific dataset [6].

In this manuscript, we present how to construct, interpret, and use a generic nomogram for a multinomial logistic prediction model. We will first specify the multinomial regression model and then present a general approach for deriving the nomogram and accompanying scoring chart for such models irrespective of the number of outcome categories that are present. We will illustrate the use of the nomogram and its interpretation using a clinical example on the risk of operative delivery [7].

## Multinomial logistic model

Let *y*_*i*_ denote the single observed outcome category of individual *i*. Assuming that this outcome is in one of *K* categories (e.g., a disease among *K* possible diseases), we may assume *Y*_*i*_ to be a multinomial random variable with probabilities *π*_*i*1_,…,*π*_*iK*_. Conditional on *J* observed covariate values in vector ***x***_*i*_, ***x***_*i*_={*x*_*i*1_,…,*x*_*ij*_,…,*x*_*iJ*_}, the probability of observing category *k* is denoted by *π*_*k*_(***x***_*i*_). The multinomial logistic model where category *K* is treated as the reference category, can then be defined as

1 $$  \ln\frac{\pi_{k}(\boldsymbol{x}_{i})}{\pi_{K}(\boldsymbol{x}_{i})} = \text{lp}_{k}(\boldsymbol{x}_{i}) = \alpha_{k} + \boldsymbol{\beta}'_{k} \boldsymbol{x}_{i}, \quad k = 1,\ldots,K-1,  $$

where *α*_*k*_ is an intercept term, ***β***_*k*_={*β*_*k*1_,…,*β*_*kJ*_} is a vector of regression coefficients and lp _*k*_(***x***_*i*_) is one of *K*−1 linear predictors for individual *i*. We can now define the probability of each possible category *k* by

2 $$\begin{array}{*{20}l} \pi_{k}(\boldsymbol{x}_{i}) = \left\{\begin{array}{rl} &{\exp\left\{\text{lp}_{k}(\boldsymbol{x}_{i})\right\}}/{\left[1+\sum_{p=1}^{K-1}\exp\left\{\text{lp}_{p}(\boldsymbol{x}_{i})\right\}\right]} \\ &\quad\text{if}\;\;k = 1,\ldots,K-1 \\ &{1}/\left[1+\sum_{p=1}^{K-1}\exp\left\{\text{lp}_{p}(\boldsymbol{x}_{i})\right\} \right] \\ &\quad\text{if}\;\;k = K. \end{array} \right. \end{array} $$

## Methods: Constructing the multinomial nomogram

The nomogram we suggest is a special case of a group of nomograms that are formally known as parallel scale nomograms. Doerfler [8] outlined the parallel scale nomogram that can be constructed if a particular value can be calculated from the sum of two functions. To use this approach for multinomial logistic models, we make use of a natural logarithm transformation applied to the elements of Eq. 2, such that,

3 $$  \ln \pi_{k}(\boldsymbol{x}_{i}) = \left\{\begin{array}{rll} \text{lp}_{k}(\boldsymbol{x}_{i}) &- \ln \left[ {1+\sum_{p=1}^{K-1}}\exp\left\{\text{lp}_{p}(\boldsymbol{x}_{i})\right\}\right] \\ &\quad\text{if}\;\;{k = 1},\ldots,{K-1} \\ &- \ln \left[{1+\sum_{p=1}^{K-1}}\exp\left\{\text{lp}_{p}(\boldsymbol{x}_{i})\right\}\right] \\ &\quad\text{if}\;\;k = K. \end{array}\right.  $$

To simplify notation, in the following we let *o*_*ik*_= ln*π*_*k*_(***x***_*i*_), *l*_*ik*_=lp_*k*_(***x***_*i*_) and $s_{i} = - \ln \left [ {1+\sum _{p=1}^{K-1}}\exp \left \{\text {lp}_{p}(\boldsymbol {x}_{i})\right \}\right ]$.

The parallel scale nomogram makes use of the relation: *o*_*ik*_=*l*_*ik*_+*s*_*i*_. Each of these three elements in this relation corresponds to one of the three vertical axes of the nomogram. The axes are denoted by *L* (left axis), *O* (middle axis), and *S* (right axis). Axis *L* is a scaled function of linear predictor *k*,*m*_1_ *l*_*ik*_, where *m*_1_ is the scaling factor. Axis *O* corresponds to the probability of observing category *k*,*m*_2_ *o*_*ik*_. Lastly, axis *S* is a scaled function of the sum of exponentiated linear predictors, *m*_3_ *s*_*i*_. Axes *O* and *S* are on the natural log scale.

**Fig. 1:**
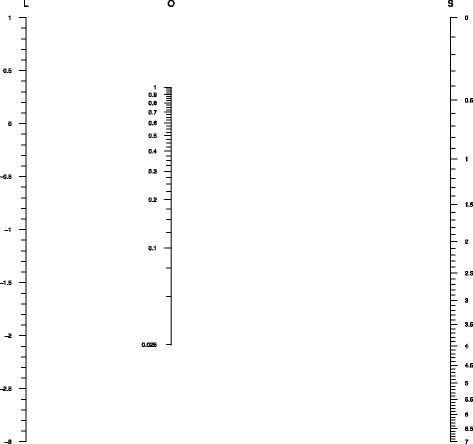
Generic nomogram for reporting multinomial logistic regression analysis. *Axis L*: lp_*k*_(x_*i*_), *Axis O*: probability of outcome *k*, *Axis S*: $\sum _{p} \exp \{\text {lp}_{p}(\mathrm {x}_{i})\}$

The nomogram is depicted in Fig. 1. Below we detail the four-step procedure to arrive at this nomogram. For further details about the construction of the parallel scale nomogram, we refer to Doerfler [8].

### Step 1: placing the outer axes (*L* and *S*)

To obtain an adequately sized nomogram, determine the desired common height (*h*) for the outer two axes (*L* and *S*) and the horizontal distance between them (*d*). The two parallel axes are placed in the vertical direction. The values for *h* and *d* are assigned at the discretion of the researcher in a common metric (e.g., centimeters or inches). Larger values for *h* and *d* will allow for more precise reading of values.

### Step 2: determine the scaling factors ***m***_***1***_, ***m***_***2***_, ***m***_***3***_

For determining the scaling factors for the outer axes (*m*_1_ and *m*_3_), the relevant ranges of *l*_*ik*_ and *s*_*i*_ need to be considered. The limits of these ranges (*l* ^low^,*l* ^up^,*s* ^low^,*s* ^up^) may be determined by the range observed in the data set where the model was developed, e.g., $l^{\text {low}} = \text {min}(\hat {l}_{ik})$ and $l^{\text {up}} = \text {max}(\hat {l}_{ik})$. These limits define the corresponding limits of the axes. Once these ranges are chosen, the scaling factors *m*_1_ and *m*_3_ are computed by *m*_1_=*h*/(*l* ^up^−*l* ^low^) and *m*_3_=*h*/(*s* ^up^−*s* ^low^). The remaining scaling factor is given by *m*_2_=*m*_1_ *m*_3_/(*m*_1_+*m*_3_).

### Step 3: placing the middle axis (*O*)

The *O* axis is placed parallel to the outer axes. The horizontal distance between the axes *L* and *O* is given by *d*_*LO*_=*d*−*d*/(*m*1/*m*3+1).

### Step 4: placing tick marks and labels

For the outer axes *L* and *S*, two sequences of values for the tick marks and corresponding labels are defined: *l*_*T*_=(*l* ^low^,…,*l*_*t*_,…,*l* ^up^) and *s*_*T*_=(*s* ^low^,…,*s* ^*t*^,…,*s* ^up^). Tick mark *t* on the axis should be placed relative to the lower end of that axis at a distance of *m*_1_×(*l*_*t*_−*l* ^low^) for axis *L* and *m*_3_×(*s*_*t*_−*l* ^low^) for axis *S*. For axis *O*, we define: *o* ^low^= exp{*l* ^low^+*s* ^low^}. Because the axis *O* represents a log transformed probability scale, first a sequence of arithmetic probabilities $o^{*}_{T}$ is defined with values between exp{*o* ^low^} and 1. Then, tick mark *t* for this sequence may be placed at $m_{2} \times (\ln (o^{*}_{t}) - f(o^{\text {low}}))$ labeled by $o^{*}_{T}$. Axis *S* is labeled by $\sum _{p=1}^{K-1}\exp \{\text {lp}_{p}(\boldsymbol {x}_{i})\}$.

## Methods: Constructing the scoring chart

The use of the nomogram by health professionals can be improved by additionally presenting a scoring chart. This scoring chart provides a graphical approach to arriving at the values for the two outer axes *L* and *S* of the nomogram for any relevant combination of values on the covariates (***x***_*i*_). For brevity, in this section we only consider the case of a multinomial logistic regression model with first order main effects. The scoring chart (and nomogram) can be extended to accommodate situations where higher order and interaction effects are present.

To make the scoring chart user-friendly, the individual effects of covariate *j*, ($\hat {\beta }_{jk}x_{ij}$), that make up the linear predictor *k* are rescaled to a “standardized” score. The sum over these individual effects together with a baseline score make up a “standardized” total score. This total score is a linear transformation of *l*_*ik*_. To facilitate the applicability of this standardized total score approach to the nomogram, the scaling of axis *L* should be adjusted accordingly. Below we detail the three-step procedure to arrive at the scoring system that makes up the scoring chart.

### Step 5: standardized covariate effects: points

The estimated multinomial logistic regression coefficients, $\hat {\beta }_{jk}$, are rescaled relative to the largest (conditional) covariate effect on a scale that has a minimum of 0 and a maximum of 100. First, the relevant ranges for each of the covariate variables are considered. Let the boundaries of these relevant ranges be denoted: ${x}^{\text {low}}_{j}$ and ${x}^{\text {up}}_{j}$. The rescaling factor and rescaled coefficients are then computed by $r = 100/{\smash {\max _{j,k}}}(| \hat {\beta }_{jk} {x}^{\text {up}}_{j} - \hat {\beta }_{jk} {x}^{\text {low}}_{j}|)$ and $\hat {\beta }^{*}_{jk} = r \times \hat {\beta }_{jk}$. The covariate effects are “standardized” by $\text {Points}_{jk}(x_{ij}) = \hat {\beta }^{*}_{jk}x_{ij} - \min (\hat {\beta }^{*}_{jk}{x}^{\text {up}}_{j},\hat {\beta }^{*}_{jk}{x}^{\text {low}}_{j})$.

### Step 6: standardized total effect: total points

A baseline score for each category (except the reference category) is defined that takes into account the standardization that has been performed at step 5. The baseline score is computed by $bl_{k} = r \times \hat {\alpha }_{k} + \sum _{j} \min (\hat {\beta }^{*}_{jk}{x}^{\text {up}}_{j},\hat {\beta }^{*}_{jk}{x}^{\text {low}}_{j})$. To also obtain a “standardized” baseline score such that the minimum rescaled baseline score is zero, we subtract the minimum baseline score, $bl_{k}^{*}= bl_{k} - {\smash {\min _{k}}}(bl_{k})$. The standardized total effect for category *k* given covariate values is then given by Total$_{k} = bl_{k}^{*} + \sum _{j} \text {Points}_{jk}(x_{ij})$. Notice that $l_{ik}=\text {lp}_{k}(\boldsymbol {x}_{i}) =({Total}_{k} + {\smash {\min _{k}}}(bl_{k}))/r$.

### Step 7: connecting the standardized total effects to the *S* axis

A horizontal axes representing Total _*k*_ is placed near the lower end of the scoring chart. Another parallel horizontal axis is placed: the values on this axis are related to the former axis by $\exp \{({Total}_{k} + {\smash {\min _{k}}}(bl_{k}))/r\}$. Taking the sum over the values that can be read off from the axis for all categories (except the reference category) is all the information necessary for determining the value on the *S* axis.

## Result and discussion

### Empirical example: predicting the risk of operative delivery

To illustrate the suggested scoring chart and nomogram for the reporting of multinomial logistic models, we use a previously published model on predicting the risk of operative delivery [7]. We detail their use by considering a specific hypothetical subject (described below). The prediction model used in this illustration was developed using data from a randomized clinical trial conducted in the Netherlands [9].

In brief, the multinomial prediction model was developed in 5667 laboring women with high-risk vertex (i.e., babies in a normal position in the uterus) singleton pregnancies beyond 36 weeks of gestation that met the inclusion criteria of the randomized clinical trial. Based on the combination of the intervention (i.e., instrumental vaginal delivery (IVD) or caesarean section (CS)) and the indication for the intervention (i.e., fetal distress (FD) or failure to progress (FTP)), women were assigned to one of five distinctive outcome categories: spontaneous vaginal delivery (reference category); instrumental vaginal delivery due to suspected fetal distress (IVD-FD); caesarean section due to suspected fetal distress (CS-FD); instrumental vaginal delivery due to failure to progress (IVD-FTP); or caesarean section due to failure to progress (CS-FTP). The multinomial regression model included the antepartum variables: maternal age, parity, gestational age, maternal diabetes mellitus, previous caesarean delivery, fetal gender, maternal hypertensive disorder, suspected intrauterine growth restriction, and antepartum estimated fetal weight. An antepartum prediction model was developed using this set of variables (i.e., model 1 in Schuit et al. 2012; see Table 1). For more details on the various outcome categories and candidate predictors, we refer to the original publication [7].

**Table 1:** Multivariable associations for multinomial antepartum prediction model, predicting the risk of operative delivery

	IVD-FD vs spont.		CS-FD vs spont.		IVD-FTP vs spont.		CS-FTP vs spont.	
	$\hat {\beta }_{jk}$	OR(95% CI)	$\hat {\beta }_{jk}$	OR(95% CI)	$\hat {\beta }_{jk}$	OR(95% CI)	$\hat {\beta }_{jk}$	OR(95% CI)
Intercept	−13.1		−15.6		−11.1		−15.4	
Maternal age, years	0.029	1.03 (1.01, 1.05)	0.052	1.05 (1.02, 1.09)	0.054	1.06 (1.03, 1.08)	0.056	1.06 (1.04, 1.08)
Gestational age, weeks	0.26	1.29 (1.18, 1.41)	0.32	1.38 (1.22, 1.56)	0.038	1.04 (0.95, 1.13)	0.13	1.14 (1.05, 1.24)
Nulliparous	2.05	7.79 (5.26, 11.5)	1.13	3.09 (2.09–4.55)	3.39	29.7 (17.2–51.1)	2.65	14.1 (9.78–20.3)
Previous caesarean delivery	1.77	5.87 (3.70, 9.32)	1.06	2.88 (1.74, 4.76)	2.39	10.9 (5.92, 20.1)	2.23	9.34 (6.17, 1.41)
Neonatal female gender	−0.19	0.83 (0.67, 1.03)	−0.5	0.61 (0.45, 0.83)	−0.25	0.78 (0.63, 0.96)	−0.013	0.99 (0.81, 1.20)
Birthweight, 100-g increments	−0.059	0.94 (0.92, 0.97)	−0.079	0.92 (0.89, 0.96)	0.083	1.09 (1.06, 1.11)	0.12	1.12 (1.10, 1.15)
Maternal diabetes mellitus	0.32	1.37 (0.65, 2.91)	0.99	2.69 (1.29, 5.60)	−0.24	0.79 (0.35, 1.76)	0.87	2.38 (1.44, 3.95)

**Fig. 2:**
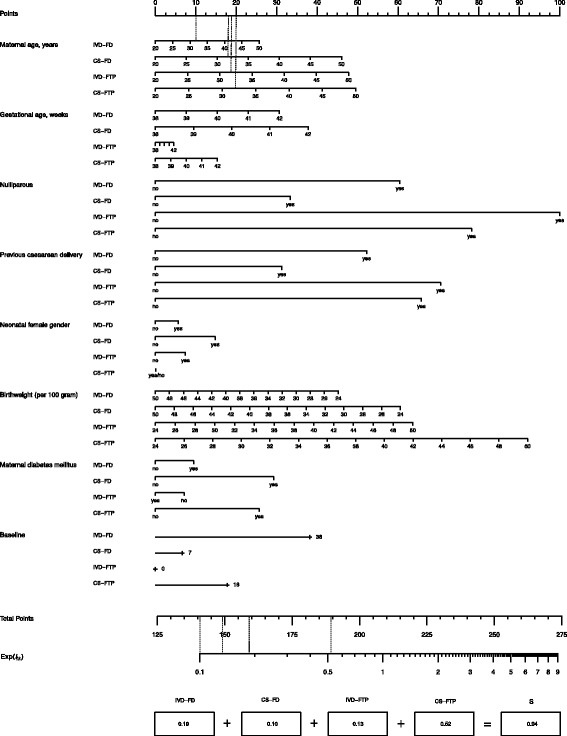
Scoring chart—hypothetical case study based on multinomial prediction model in Schuit et al. Case description: Maternal age: 32 years; gestational age: 40 weeks; nulliparous; birth weight: 3540 g, maternal diabetes. Abbreviations: instrumental vaginal delivery (IVD), caesarean section (CS), fetal distress (FD), failure to progress (FTP)

**Fig. 3:**
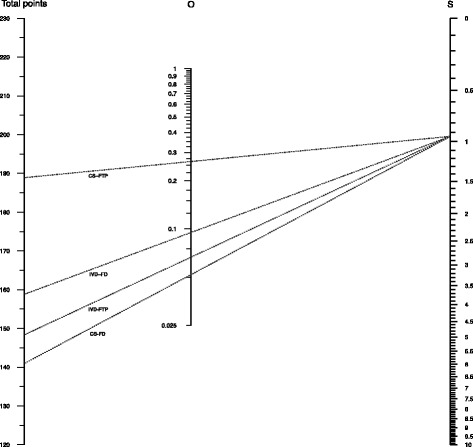
Nomogram—hypothetical case study based on multinomial prediction model in Schuit et al. Case description: Maternal age: 32 years; gestational age: 40 weeks; nulliparous; birth weight: 3540 g, maternal diabetes. Abbreviations: instrumental vaginal delivery (IVD), caesarean section (CS), fetal distress (FD), failure to progress (FTP)

Consider the following hypothetical subject: a nulliparous diabetic subject with a maternal age of 32 years, a gestational age of 40 weeks, expecting a boy with an estimated birth weight of 3540 g. Using the results presented in Table 1 and a series of calculations (that follow from Eqs. 1 and 2), one may calculate the predicted probabilities for the hypothetical case study for each of the five outcomes as 0.096 (IVD-FD), 0.051 (CS-FD), 0.069 (IVD-FTP), 0.268 (CS-FTP), and 0.516 (spontaneous delivery). To facilitate the calculation of the predicted probabilities, we constructed a scoring chart (Fig. 2) and nomogram (Fig. 3) using the reported regression coefficients by Schuit et al. [7]. For illustrative purposes we have added some information specific to the case study in Figs. 2 and 3. The scoring chart and nomogram without the case study information can be found in Webfigure A and Webfigure B, respectively.

Using the scoring chart (Fig. 2), each subject characteristic can be converted into a score for a particular outcome category (except the reference category). We have illustrated this procedure by drawing vertical dashed lines between the axes only for the subject characteristic “maternal age” (32 years) to the Points axis (upper end scoring chart) in the figure. We leave it to the interested reader to draw the lines for the remaining characteristics. By adding the points of the separate predictors to the baseline points for each of the chosen outcomes, one obtains the total points for this particular outcome. Each of these total points should be marked at the line indicated by Total Points (lower end scoring chart). For the considered subject, the total points are 159 (IVD-FD), 140 (CS-FD), 149 (IVD-FTP), and 189 (CS-FTP). Further, by drawing vertical lines from the marked points on Total Points axis to the Exp(*l*_*ik*_) axis (illustrated using dashed lines), one can read-off the exponentiated linear predictor for each of the outcome categories. This information is then put in the boxes right under the Exp(*l*_*ik*_) axis. Finally, by taking the sum over these values (registered under box “S”) one obtains all information needed to use the nomogram (Fig. 3).

To use the nomogram (Fig. 3), one first marks on the Total Points axis (left axis) the total points for each outcome category except the reference category, as derived from the scoring chart. One than marks the value registered under box “S” in the scoring chart on the *S* axis of (Fig. 3). The points marked on the Total points axis and point on the *S* axis are then connected using straight lines. From the middle “*O*” axis, one can now read-off the predicted probabilities for each of the four outcomes for this particular case. Finally, by subtracting these four probabilities from one, the probability of the reference category is obtained, corresponding to the probability of a “spontaneous delivery”.

## Conclusion

Nomograms are not likely to be used very often in contemporary clinical practice for calculating probabilities for individual patients given the considerable burden placed on the user and also because its accuracy is limited by the precision with which physical markings can be drawn, reproduced, viewed, and aligned. For this purpose, electronic implementation of a prediction model by means of a website calculator, mobile app, or an algorithm implemented into the electronic medical file of the patients to directly calculate the relevant probabilities may be considered. However, we still think that nomograms remain quite useful, even today, as they do offer great visual insight a (multinomial) prediction model, which is often perceived as a black-box (for a further discussion on the usefulness of nomograms we refer to [10, 11]). Therefore, we do expect that our general approach to construct and report scoring charts and nomograms for multinomial logistic regression models will facilitate the interpretation and use of such models to a wider audience. Our approach is flexible and generalizable and can be used irrespective of the number of outcome categories and types of covariates present. R-code for developing the nomogram is available from http://mvansmeden.net/software/multinomial-nomogram.

## Abbreviations

CS: Caesarean section
FD: Fetal distress
FTP: Failure to progress
IVD: Instrumental vaginal delivery
